# Nutritional Attributes and Sensory Quality during Storage Time of Spiced Lamb Burgers from Manchego Spanish Breed

**DOI:** 10.3390/foods9101466

**Published:** 2020-10-15

**Authors:** María Belén Linares, Almudena Cózar, María Dolores Garrido, Herminia Vergara

**Affiliations:** 1 Veterinary Faculty, Food Science and Technology Department, University of Murcia, 30100 Espinardo-Murcia, Spain; blinares@um.es (M.B.L.); mgarrido@um.es (M.D.G.); 2 Science and Agroforestry Technology and Genetic Department, University of Castilla-La Mancha, Higher Technical School of Agricultural Engineering and Forestry, 02071 Albacete, Spain; Almudena.Cozar@uclm.es; 3 Food Quality Section Albacete, Regional Development Institute, University of Castilla-La Mancha, 02071 Albacete, Spain

**Keywords:** lamb, spiced-burgers, different-meat-cuts, sensory-chemical-properties

## Abstract

The aim of this study was to determine the effect of the addition of spices (garlic, rosemary, thyme, sage or clove) on the chemical and sensory characteristics of burgers elaborated with lamb meat from different locations (L: only leg; LNB 2/3 leg + 1/3 neck and breast). Formulation type (L vs. LNB) modified the chemical composition. L burgers showed lower values of fat, saturated fatty acids, monounsaturated fatty acids and higher moisture and polyunsaturated fatty acids content than LNB samples, with both types of burgers maintaining the nutritional indexes (P/S and n6/n3) within the recommended amounts. Nevertheless, the type of formula did not influence the sensory properties of lamb burgers. By contrast, the spice addition affected these characteristics over the period of time considered in this study. At the end of storage their global quality could be listed in the following order, from major to minor rate: Sage > Rosemary > Garlic > Thyme > Clove burgers.

## 1. Introduction

Sheep are traditionally raised in the most marginal areas of Mediterranean Europe considered mainly as High Nature Value (HNV) farmland [1]. The lamb meat industry is focused on primary transformation, thus, the most important challenge for the industry is based on technological innovation and use of lamb-based new product development [2]. Differentiation and specificity are the two basic aspects to be considered in the meat industry, but few studies focused on the lamb meat market diversification, since meat from other species, such as pork or beef, has tended to dominate the market [3]. In this sense, while a decrease in lamb meat consumption has been observed (average of 1.57 kg per person and year in Spain [4]), some authors [5,6,7] have pointed out that the creation and innovation of quality products could be a key strategy to reverse this situation. A study carried out in Spain, showed that lamb meat is not a product usually consumed by young people (<25 years) [2] and in this sense “lamb burgers” could be a format product highly appreciated by this sector-age population. In addition, in our previous work [6], the possibility of using pieces of lamb carcass of little commercial value to make meat products, such as hamburgers, obtaining quality product labelling under a Protected Geographical Indications (PGIs), was highlighted. This could lead manufacturers to present their products under different labels in order to offer value-added differentiation and ensure the entry into a given markets at lower prices [8]. For the usual lamb meat consumers, mainly convenience with new recipe possibilities and diversified diets are the most important topics considered. Nowadays, other topics as the health benefits, ethical and social implications are the new criterions considered by consumers at the purchase time [9]. Meat can be classified as “good experience” meaning that its sensory attributes, tenderness and flavour, are probably one of the most decisive factors at purchase time [10,11]. On the other hand, eating habits have changed in the last years, the European consumers demand foods with quick and simple preparations [12]. Therefore, is necessary to update the form of lamb meat consumption with the development of new products (lamb burgers) that could expand the markets for selling them, such as the Halal or Kosher (with the necessary adaptations to preserve their requirements), which are starting to insert in the Spanish market [13]. Moreover, globalization has evolved the way of cooking, introducing new recipes and seasonings in the traditional cuisines of each country [14]. In this sense, in recent years, the use of spices [15] due to the trend towards the consumption for natural products without synthetic additives in their formulations [16] is demanded, providing meat products with their characteristic aroma, colour, flavour, taste or texture [17]. Furthermore, it is well known that an assessment of sensory characteristics is crucial in developing of new products and markets [18]. Many studies have pointed out the effect of spices in meat and meat products [16,19,20,21,22,23] but the sensory profile of spiced lamb burgers as a new product range has not so highly evaluated [24,25], despite the fact that some natural plant extracts added to lamb patties have shown an important protective effect during storage [5,7,26,27,28,29]. The goal of this study was to evaluate the effect of the formulation type, made with leg meat (L) or leg + neck + breast (LNB) meat, and powered spiced addition (garlic, rosemary, thyme, clove or sage) on the nutritional attributes (proximate composition, fatty acid profile and healthy indexes) and sensory profile of cooked spiced lamb burgers at 0, 3 and 6 days of storage in order to try to revalorize lamb cut from the Manchego breed labelled under the PGI.

## 2. Materials and Methods

### 2.1. Preparation of Burgers

Burger meat was obtained from the meat of legs, necks and breasts of Spanish Manchega breed lamb carcasses (*n* = 11) belonging to the official label “Manchego Lamb PGI”. Animals were fed with milk until 12 kg of weight and cereal straw ad libitum was administered until slaughter time at 25 kg live weight (70 days old). After slaughtering and dressing by standard commercial procedures, all carcasses were chilled at 4 °C for 24 h in a conventional chiller [6]. These meat pieces were deboned and in order to prepare two formulas of burgers, meat leg was minced separately from the remaining pieces (neck and breast). In addition, these two later pieces were minced together. So, two types of burgers were made according to the meat used to elaborate:

L burgers: completely made from hindleg lamb minced meat.

LNB burgers: elaborated using 2/3 hindleg lamb minced meat and the rest (1/3) neck and breast.

After being minced, both types of meat products (L and LNB) were divided in five batches, to which were added salt (1%, *w*/*w*) and a powdered spice (0.1%, *w*/*w*). The spices were purchased at supermarket and ground in the laboratory before the addition. We obtained garlic and clove (Drucos, Barcelona, Spain), rosemary and thyme, (Artemis, Alicante, Spain) and sage (Soria Natural, Soria, Spain) for each type of formulation (L and LNB). Then, the batches were mixed by hand 5 min for uniform distribution. Afterwards, burgers were shaped using a commercial burger maker (model 2R, BECAM, Jose Bernad S. L., Albacete, Spain). From each batch two replicates were made in different days (L, *n* = total 80; *n* = 40 each replicate; LNB, *n* = total 80, *n* = 40 each replicate, respectively). Once the burgers were made, they were packed in white expanded polystyrene trays (Model 90 m, Coopbox Hispania S.L.U, Lorca, Murcia, Spain) and covered with a polyvinyl chloride (PVC) film with an oxygen permeability of 12,500–13,200 cm^3^/m^2^ per 24 h and water vapour permeability of 200–230 g/m^2^ per 24 h (Coopbox Hispania S.L.U, Lorca, Murcia, Spain) under aerobiosis conditions and storage at 2 °C for 6 days. The analysis controls were made at 0, 3 and 6 days after packing. Burgers were packed under vacuum conditions at these analysis days and frozen at −20 °C in a freezer until the analysis was carried out. The study of the batch control (non-spiced, only with 1% of salt) of each formulation (L vs. LNB) was realized and presented in our previous work [6].

### 2.2. Proximal Composition

The standard methods [30] were used to analyse the moisture, protein, ash and fat contents. Moisture % was evaluated by desiccation in an oven at 105 °C 24 h; Protein was analysed with the Kjeldahl method, in a digestor block Digestion Unit Buchi 426 (Büchi labortechnik AG, Flawil, Switzerland); fat content was determined through the Soxhlet method, in an unit extraction system Buchi B-811 (Büchi labortechnik AG, Flawil, Switzerland); Ash percentage was obtained after 4 h at 550 °C, in an oven with a temperature slope.

### 2.3. Fatty Acid Composition

The fatty acid profile was analysed according to the [31] procedure. Meat samples (10 g) were homogenized with 200 mL of chloroform–methanol (2:1 *v*/*v*) solution for 3 min. After blending, 50 mL of chloroform and 80 mL of a 10% aqueous NaCl solution were added to separate the methanol-water phase. After 12 h the lipid extracts were collected, and the solvents evaporated (Rotavapor R-200 Buchi, Büchi Labortechnik AG, Flawil, Switzerland). Fatty acids were converted to methyl esters with sodium methylate (0.2 N) in an acid medium at 3% (sulfuric acid in anhydrous methanol). After methyl esters were analysed using a gas chromatograph (Hewlett Packard 6890 N, Agilent Technologies, Folsom, CA, USA) fitted with a flame ionization detector and quantified using undecanoic acid as an internal standard. Samples (1 µL) were injected in a capillary column HP 5 (30 m length, 0.32 internal diameter and 0.25 µm thickness). After injection, the initial column temperature of 80 °C increased to 155 °C at 3 °C/min. Then was increased to 205 °C at 5 °C/min, and finally increased to 206 °C at 0.1 °C/min maintained for 15 min. The total time of analysis was 60 min. Temperatures for the injector and the detector were 280 and 300 °C, respectively. The carrier gas was helium (3.2 mL/min). Fatty acid identification was based on the comparison with the retention times of peaks from samples with standards.

### 2.4. Sensory Evaluation

For the sensory analysis, the burgers, previously frozen at −24 °C at 0, 3 and 6 days post-packing, respectively, were thawed at 4 °C overnight and then cooked between two heating plates covered with aluminium foil (Silanos, Liscia Average, Lavastoviglie Industriali, Italy) at 150 °C for six minutes, reaching an internal temperature of 72 °C, as measured by a portable T200 thermometer (Digitron Instrumentation Ltd., Merd Lane, Hertford, United Kingdom). Rectangular pieces of approximately 1.5 × 2 cm from the centre of the burgers were obtained and covered immediately with aluminium foil. Samples were kept at 60 °C in sand baths (Braun, Espluges de Llobregat, Barcelona, Spain) until they were presented to the panellists in a balanced order [32].

The panel was formed of eight trained assessors in the profile assessment of different meat products chosen from the staff of the University of Murcia. They were trained according to [33]. Seven training sessions were carried out: in the three first, descriptors of raw/cooked lamb burgers were studied, and the following four sessions were concerned with identifying, selecting and quantifying attributes to evaluate the burgers.

During training the panel was provided with samples of freshly cooked lamb burgers (as reference for optimal values of the sensory characteristics) and cooked burgers stored under refrigeration for long periods of time (as reference of the attributes associated with deteriorated cooked burgers). A seven-point scale was established using other foods or sensory standards as reference. In training and panel performance sessions, samples were coded with random three-digit numbers. Mineral water was provided for mouth rinsing between samples. Sensorial analysis was carried out according to [34] using a linear scale of 1 (minimum) to 7 (maximum). The descriptors used were: Meat Odour (MO); Spiced Odour (SO); Rancid Odour (RO); Meat Colour (MC); Meat Flavour (MF); Spiced Flavour (SF); Rancid Flavour (RF). In addition, the Global Quality was evaluated (GQ: 1 = minimum to 7 = maximum). Each panellist examined by triplicate all types of burgers (L: garlic, rosemary, thyme, clove or sage. LNB: garlic, rosemary, thyme, clove or sage)/three storage times post-packing (0, 3, 6 days).

### 2.5. Statistical Analysis

Data were analysed with the SPSS version 22 statistical package [35]. The effect of the formulation type (L: leg meat vs. LNB: 2/3 leg meat and 1/3 neck + breast) and the differences on nutritional attributes (moisture, protein, ash and fat), fatty acid profile and spice added to burgers (rosemary, thyme, sage, garlic or clove) was analysed using an analysis of variance (ANOVA). The sensory test was evaluated with an ANOVA to study the effect of spices added (rosemary, thyme, sage, garlic or clove) and when the differences were significant (*p* < 0.05) a Tukey’s test at a significance level of *p* < 0.05 verified the differences between pair of groups. A general linear model procedure was used to examine the effect of the type of meat and the types of spices on burgers sensory quality. For each group of burgers the differences in the sensory quality attributes were calculated to study the effect of spice, formula and the interaction spice*formula (*p* < 0.05).

## 3. Results

### 3.1. Chemical Characteristics and Healthy Indexes

Table 1 shows the proximal composition of spiced lamb burgers from two different formulations (L: Leg meat; LNB: 2/3 leg + 1/3 neck and breast meat). In all the groups considered, a higher fat and a lower moisture contents were observed (*p <* 0.001) on the LNB burgers than L samples. In general, no significant effect of formulation (L vs. LNB) was obtained for the protein and ash content. In general, the LNB burgers presented (Table 2) a higher level of Total Saturated Fatty Acids (SFA), Total Monounsaturated Fatty Acids (MUFA) (except in rosemary and clove samples), Saturation index SFA/PUFA (S/P) and Thrombogenic Index (TI) than L burgers. In contrast, L samples showed higher values (*p* < 0.001) of Total Unsaturated Fatty Acids (UFA), Total Polyunsaturated Fatty Acids (PUFA), and PUFA/SFA (P/S) than LNB formulation. The spice type did not affect the chemical composition or healthy indexes.

### 3.2. Sensory Analysis in Spiced Lamb Burgers

The effect of type of powdered spice used on sensorial attributes of lamb burgers is showed in the Table 3 (Type L) and Table 4 (Type LNB). In general, the spices added presented statistical differences in all sensorial attributes studied, except in L burgers on rancid odour and flavour (at 0 d) and in LNB burgers on rancid odour and flavour (in all period of study) and meat colour (at 3 d). The L and LNB burgers with clove presented the lowest values of meat odour and flavour and the highest scores of spiced odour and flavour on all the days of analysis. The general linear model (Table 5), considering as factors, the spice (rosemary, thyme, sage, garlic or clove) and formulation type (L vs. LNB) confirmed that the addition of spice had a significant effect on sensory analysis of lamb burger (*p <* 0.001), the formulation did not affect the attributes evaluated and a significant interaction between factors (Batch*Formula) on meaty (*p <* 0.05), rancid odour (*p <* 0.001) and spiced and rancid flavour (*p <* 0.05) was demonstrated.

Global Quality (GQ) of cooked spiced lamb burgers from both formulations (L vs. LNB) showed that clove samples reached the lowest scores (around 2–3 point) in all the period of study. On the other hand, L and LNB samples with sage and rosemary remained stable in the time with a high score (around 5), showing the burgers with thyme values around 4. The garlic LNB burgers had a high score (around 5) during storage period, but the L samples with garlic decreased (data not shown).

Formula L: Leg lamb meat. Formula LNB: 2/3 leg + 1/3 neck and breast meat. **, ***: Significance level of 0.01 and 0.001, respectively. NS: not significant.

## 4. Discussion

### 4.1. Chemical Characteristics and Healthy Indexes

Meat and meat products contain fat, in different amount and profiles types, which could affect human health if they are ingested in high amounts [36]; therefore, it is crucial to know the chemical composition and health indexes for development of new products with these desirable characteristics.

Overall, the use of different pieces of carcass has not affected the content of protein with respect to ash content, and [37] noted that goat and sheep sausages with higher fat level presented lower ash content.

According to [38], the content of fat in meat and meat products is influenced, among other factors, by the anatomical part of the carcass used and this parameter has an inverse relationship with moisture [39]. This fact could explain the statistical differences found in the fat level among L and LNB, with LNB (2/3 leg + 1/3 neck and breast) burgers showing a higher fat content and lower values of moisture than L (only with leg) samples. Others like [7] noted a lower fat content (2.3%) in lamb burgers elaborated with leg meat of Merino breed, slaughtered at 24 kg live weight, and a moisture proportion slightly higher. Some authors [25] found higher values of fat (22.62%) in lamb burgers elaborated with neck and fat trimmings of lamb without specifying the breed. These differences in fat and moisture content among the studies could be due to the breed used and the final live weight before slaughter of animals [38] along with previous meat preparation (fat removal) to the elaboration of burgers [40].

The different pieces included in the carcass could show differences in the quantity and components of the fat [41]. The fatty acid profile presented a remarkable influence by the formula (L vs. LNB), with and lower content of SFA and MUFA and higher PUFA values in the L burgers than LNB burgers. In addition, the fat content and composition are directly related to lipid oxidation, within the fatty acid the UFA and mainly the PUFA showed a tendency to oxidation process over time [42].

In our study, all types of hamburgers had P/S and n6/n3 values within nutritional recommendations (between 0.4–1.0; <4; respectively) [43,44], and these indexes were lower than obtained in other meat products analysed in other studies [45]. Other authors [46] noted that the index P/S did not consider the effect of MUFA and took into account that all SFAs were affected in the same way in terms of the cholesterol increase. In this sense, other nutritional indexes such as atherogenic (AI) and thrombogenic (TI) could indicate the ability of atherogenicity or thrombogenicity of foods [47] and lower values of these indexes indicate a reduction of possible vascular risk [48]. In general, all of the samples had lower values of AI and TI than observed by [49] in spiced rabbit burgers with ginger or non-spiced.

Spices have been considered a good source of micronutrients [50]. However, in the present study, any significant difference was observed between control and the spiced groups. Many spices have been used in meat and meat products in order to minimize the oxidant and spoilage effect from the storage time [51]. Thus the shelf –life and organoleptics qualities improvements could also allow enhanced nutritional benefits but related with flavonoids and phenolics compounds. Since these spices are not added in large quantities the contribution on the nutritional indexes are not significant [52].

### 4.2. Sensory Analysis in Spiced Lamb Burgers

The herbs and spices could have several benefits; firstly, their use could provide special and new taste and aroma in foods and enhances their flavour, which could diversify the product offer and their addition could decrease the use of salt and sugar in these foods [17]. On the other hand, these natural substances could help to maintain and improve the quality and shelf life of these perishable products and might avoid the utilization of synthetic preservatives, due to their antioxidant and antimicrobial properties, without forgetting their medicinal effects [15].

The addition of powdered spices showed significant differences in both formulations during time of study. When compared the spiced samples with control burgers at the beginning of study (data of control samples showed in [6], in both types of formula, the control samples presented the highest MO and MF, but over time the RF and RO scores increased in these control samples. By contrast, these rancidity attributes were relatively stable in the spiced burgers. Other authors [53] related inversely the spiced and meat flavour in ground beef with different spices (clove, cinnamon, fennel, pepper, star anise or retail 5-spice), and these spiced samples presented a low flavour of meat and the rancidity was barely appreciated by a trained panel due to the addition of this substance.

It is pointed out by [6] that some of the fundamental attributes for the GQ of lamb burgers are the rancid odour and flavour. In this study, all cooked spiced burgers showed lower scores of RO and RF than the burgers control (data showed in [6]), except the garlic burgers, which showed the highest values of lipid oxidation (3.4–3.9 mg MDA/kg^−1^ of meat, L and LNB respectively) along with the control samples (2.63–3.03 mg MDA/Kg of meat, L and LNB respectively) [27] and these lipid oxidation values did not show statistical differences among formula (L or LNB). It is well known that these undesirable attributes are related to lipid oxidation [16].

Furthermore, [19] noted in packed lamb steak that off-odours were perceived in control lamb steak when the lipid oxidation values above 2 mg MDA/Kg of meat. However, the highest levels of Thiobarbituric Acid Reactive substances (TBARs) of garlic samples in raw lamb burgers only were slightly reflected in the RO and RF attributes of L cooked products from 3 days of storage. This fact shows the capacity of the garlic to mitigate odour and the flavours of rancidity. It was found by [54] that addition of garlic or a mixture of garlic and onion was able to cover up the characteristic odour of irradiated meat in ground beef, this fact could be due to the sulphur compounds unique of garlic, and these compounds could be the reason why the judges did not notice the RO and RF in these samples with garlic.

On the other hand, in some cases the consumers could consider the pungency and spiced flavour characteristic of garlic as unpleasant [54]. However, in this study the addition of garlic showed lower values of SO and SF, and even though the GQ values of garlic burgers decreased with the time of storage, these scores were higher than those obtained in thyme, clove and control samples (showed in the previous paper [6]).

By contrast, although this concentration of clove (0.1%) obtained excellent results to maintain the lipid oxidation under control [27], these lamb burgers in both formulations presented the lowest values of GQ and MO, MF, and the highest scores of SO and SF over the duration of the study. Other authors also found high scores of spiced flavour in ground beef crumbles with powdered clove (0.1% of concentration), providing a strong flavour to ground beef according to the panellists [53].

Regarding meat colour, the sensorial property of this work was stable in all of the study period in spiced lamb burgers with scores around 5. Only the clove samples showed lower values of colour but without changes over time; however, control samples presented a loss of colour over time, mainly in L formula (data showed in [6]). Other authors [20] have noted a slight discoloration over the period of study in cooked spiced pork patties with clove, rosemary, cassia bark and control (non-spiced).

In general, the global quality of spiced lamb burgers could be listed in the following decreased order: Sage > Rosemary > Garlic > Thyme > Clove at 6 days of storage. Other authors noted a higher acceptance general for lamb burgers with antioxidants (different dose of oregano extract and sodium erythorbate) than control burgers, but there were no differences between synthetic and oregano extract [25]. It was also noted by [24] that the addition of spices (oregano and Melissa extracts) did not showed differences among them, and the acceptance scores were near to 7 “like moderately”.

Finally, despite the different composition of fatty acids and that reduction of fat could influence in the meat flavour and palatability [41,55], but in this work these differences between chemical parameters in both formulations did not affect the sensory analysis.

## 5. Conclusions

The present study showed that the addition of spices could be an alternative to diversify lamb meat products such as hamburgers, without affecting the nutritional content but considerably influencing the sensory attributes. The clove burgers presented lower scores to global quality than sage, rosemary, thyme and garlic ones, and the addition of garlic decreased the perception of rancidity. Therefore, the addition of these powdered spices in the formulation of lamb burgers developed a double action; their use contributed a pleasant odour and flavour and extended the shelf life of lamb meat products, improving sensorial attributes. On the other hand, the results obtained by use of different formulations (L vs. LNB) had no effect on the sensory properties, but the use of neck and breast in the formulation (LNB) presented higher fat and lower moisture level than L burgers. Although LNB burgers showed higher SFA and lower PUFA content than L samples, both types of formula (L and LNB) reached the nutritional indexes (P/S and n-6/n-3) within the recommendations. Therefore, the utilization of different category pieces for burger manufacturing could make it easier and more profitable to use of the whole carcass; in addition, the spices added could help to develop burgers with specific tastes, increasing the lamb products’ range in markets.

## Figures and Tables

**Table 1:** Proximal composition (%; mean ± s.e.) of spiced lamb meat burgers from two different formulation types packaged under aerobiosis conditions.

%	Formula (*n* = 80)	Garlic (*n* = 16)	Rosemary (*n* = 16)	Thymus (*n* = 16)	Clove (*n* = 16)	Sage (*n* = 16)
Moisture	L	72.51 ± 0.23	72.10 ± 0.34	72.47 ± 0.41	72.59 ± 0.84	73.32 ± 0.33
	LNB	67.79 ± 0.26	67.14 ± 0.12	66.82 ± 0.17	67.81 ± 0.19	67.07 ± 0.65
		***	***	***	***	***
Protein	L	19.94 ± 0.49	18.64 ± 0.31	18.46 ± 0.57	19.08 ± 0.56	18.39 ± 0.37
	LNB	19.94 ± 0.81	18.58 ± 0.51	18.72 ± 0.30	19.35 ± 0.26	18.20 ± 0.64
		NS	NS	NS	NS	NS
Ash	L	1.74 ± 0.02	1.92 ± 0.02	2.02 ± 0.01	1.93 ± 0.04	1.89 ± 0.02
	LNB	1.87 ± 0.05	1.82 ± 0.02	1.74 ± 0.01	1.77 ± 0.08	1.94 ± 0.02
		NS	NS	***	NS	NS
Fat	L	5.70 ± 0.27	4.68 ± 0.49	4.84 ± 0.80	5.18 ± 0.22	4.95 ± 0.43
	LNB	9.99 ± 0.30	11.47 ± 0.26	11.83 ± 0.31	10.26 ± 0.35	11.24 ± 0.56
		***	***	***	***	***

Formula L: Leg lamb meat. Formula LNB: 2/3 leg + 1/3 neck and breast meat. ***: Formula effect. Significance level 0.001. NS: not significant.

**Table 2:** Nutritional indexes (%; mean ± s.e.) of spiced lamb meat burgers from two different formulation types under aerobiosis conditions.

Fatty Acid Profile	Formula	Garlic	Rosemary	Thymus	Clove	Sage
SFA	L	45.78 ± 0.22	45.38 ± 0.27	44.31 ± 0.08	45.04 ± 0.27	44.78 ± 0.16
	LNB	47.05 ± 0.10	47.59 ± 0.10	47.39 ± 0.10	47.50 ± 0.10	47.91 ± 0.52
		***	***	***	***	***
UFA	L	54.22 ± 0.22	54.62 ± 0.27	55.69 ± 0.08	54.96 ± 0.27	55.22 ± 0.16
	LNB	52.95 ± 0.10	52.41 ± 0.10	52.61 ± 0.10	52.50 ± 0.10	52.09 ± 0.52
		***	***	***	***	***
MUFA	L	36.05 ± 0.22	35.64 ± 0.29	35.53 ± 0.12	36.39 ± 0.13	35.20 ± 0.20
	LNB	37.11 ± 0.13	36.16 ± 0.12	36.59 ± 0.11	36.64 ± 0.12	36.12 ± 0.36
		***	NS	***	NS	*
PUFA	L	18.17 ± 0.16	18.98 ± 0.52	20.16 ± 0.06	18.58 ± 0.17	20.02 ± 0.31
	LNB	15.84 ± 0.07	16.25 ± 0.14	16.02 ± 0.07	15.86 ± 0.06	15.97 ± 0.18
		***	***	***	***	***
n3/n6	L	0.54 ± 0.02	0.59 ± 0.05	0.50 ± 0.01	0.52 ± 0.01	0.47 ± 0.02
	LNB	0.54 ± 0.00	0.48 ± 0.00	0.52 ± 0.01	0.51 ± 0.00	0.54 ± 0.01
		NS	NS	NS	NS	***
n6/n3	L	1.88 ± 0.02	1.81 ± 0.11	2.00 ± 0.03	1.92 ± 0.02	2.14 ± 0.07
	LNB	1.87 ± 0.01	2.07 ± 0.01	1.92 ± 0.03	1.95 ± 0.02	1.88 ± 0.05
		NS	*	NS	NS	NS
S/P	L	0.77 ± 0.01	0.77 ± 0.01	0.73 ± 0.00	0.76 ± 0.01	0.75 ± 0.00
	LNB	0.84 ± 0.00	0.85 ± 0.00	0.85 ± 0.00	0.85 ± 0.00	0.86 ± 0.02
		***	***	***	***	***
AI	L	0.80 ± 0.03	0.75 ± 0.01	0.73 ± 0.00	0.75 ± 0.01	0.72 ± 0.01
	LNB	0.75 ± 0.00	0.75 ± 0.01	0.76 ± 0.01	0.77 ± 0.01	0.80 ± 0.03
		NS	NS	***	NS	*
TI	L	0.75 ± 0.01	0.74 ± 0.00	0.66 ± 0.00	0.72 ± 0.01	0.68 ± 0.01
	LNB	0.82 ± 0.00	0.81 ± 0.01	0.82 ± 0.00	0.82 ± 0.00	0.83 ± 0.02
		***	***	***	***	***
P/S	L	0.40 ± 0.00	0.42 ± 0.01	0.43 ± 0.00	0.39 ± 0.01	0.42 ± 0.01
	LNB	0.34 ± 0.00	0.34 ± 0.00	0.34 ± 0.00	0.33 ± 0.00	0.33 ± 0.01
		***	***	***	***	***

Formula L: Leg lamb meat. Formula LNB: 2/3 leg + 1/3 neck and breast meat. SFA, total saturated: (C10 + C12 + C14 + C16:0 + C17 + C18:0). UFA, total unsaturated. MUFA, total monounsaturated: (C16:1 + C18:1). PUFA, total polyunsaturated: (C18:2 (*n-6*) + C18:3 (*n-3*) + C20:4). *n-6/n-3*: C18:2/C18:3; *n-3/n-6*: C18:3/C18:2; S/P, saturation index: (C14:0 + C16:0 + C18:0)/ΣMUFA + PUFA; P/S: ΣPUFA/SFA; AI, atherogenic index: (C12:0 + 4 × (C14:0) + C16:0)/(ΣMUFA + Σ(*n-6*) + (*n-3*)); TI, thrombogenic index: (C14:0 + C16:0 + C18:0)/(0.5 × ΣMUFA + 0.5 × Σ(*n-6*) + 3 × Σ(*n-3*) + Σ(*n-3*)/Σ(*n-6*)). *, ***: Formula effect. Significance level of 0.05 and 0.001, respectively. NS: not significant.

**Table 3:** Effect of type of spice added on sensory attributes (mean ± s.e.) of cooked lamb meat burgers from Formula L (only leg meat) under aerobiosis conditions for up 0 to 6 days.

Attributes	Analysis Time	Garlic	Rosemary	Thymus	Clove	Sage	Effect of the Spice
**Odour**							
Meat	0	4.50 ± 0.22 ^b^	4.70 ± 0.25 ^b^	4.10 ± 0.15 ^b^	2.00 ± 0.30 ^a^	4.67 ± 0.16 ^b^	***
	3	3.40 ± 0.24 ^b^	4.55 ± 0.17 ^c,d^	3.80 ± 0.19 ^b,c^	2.03 ± 0.23 ^a^	5.00 ± 0.13 ^d^	***
	6	3.90 ± 0.18 ^b^	4.05 ± 0.22 ^b,c^	3.00 ± 0.27 ^b^	1.50 ± 0.22 ^a^	5.05 ± 0.30 ^c^	***
Spiced	0	2.90 ± 0.24 ^a^	2.30 ± 0.20 ^a^	3.15 ± 0.17 ^a^	4.85 ± 0.40 ^b^	2.17 ± 0.17 ^a^	***
	3	1.40 ± 0.40 ^a^	2.40 ± 0.22 ^b,c^	3.25 ± 0.26 ^c^	5.17 ± 0.21 ^d^	2.00 ± 0.18 ^a,b^	***
	6	2.97 ± 0.19 ^b^	3.05 ± 0.17 ^b^	3.30 ± 0.20 ^b^	5.20 ± 0.25 ^c^	1.55 ± 0.29 ^a^	***
Rancid	0	1.00 ± 0.00	1.00 ± 0.00	1.10 ± 0.10	1.05 ± 0.05	1.13 ± 0.09	NS
	3	2.40 ± 0.48 ^b^	1.05 ± 0.05 ^a^	1.30 ± 0.15 ^a^	1.00 ± 0.00 ^a^	1.10 ± 0.10 ^a^	***
	6	1.80 ± 0.19 ^b^	1.05 ± 0.05 ^a^	1.10 ± 0.10 ^a,b^	1.00 ± 0.00 ^a^	1.00 ± 0.00 ^a^	***
**Colour**							
Meat	0	6.20 ± 0.20 ^b^	5.43 ± 0.23 ^a,b^	5.40 ± 0.24 ^a,b^	4.60 ± 0.31 ^a^	5.33 ± 0.18 ^a,b^	***
	3	4.60 ± 0.53 ^a,b^	5.70 ± 0.19 ^c^	5.35 ± 0.13 ^a,b,c^	4.50 ± 0.23 ^a^	5.60 ± 0.21 ^b,c^	***
	6	5.07 ± 0.28 ^a,b^	5.60 ± 0.12 ^b^	5.60 ± 0.19 ^b^	4.20 ± 0.37 ^a^	5.60 ± 0.10 ^b^	**
**Flavour**							
Meat	0	5.30 ± 0.25 ^c^	4.33 ± 0.23 ^b,c^	3.65 ± 0.30 ^b^	2.25 ± 0.32 ^a^	4.6 ± 0.16 ^b,c^	***
	3	3.80 ± 0.34 ^b^	4.50 ± 0.18 ^b^	3.54 ± 0.22 ^b^	2.30 ± 0.32 ^a^	4.40 ± 0.19 ^b^	***
	6	4.17 ± 0.19 ^b^	4.10 ± 0.23 ^b^	3.20 ± 0.25 ^b^	1.80 ± 0.34 ^a^	4.30 ± 0.44 ^b^	***
Spiced	0	2.70 ± 0.20 ^a^	2.90 ± 0.20 ^a^	3.40 ± 0.23 ^a,b^	4.55 ± 0.44 ^b^	2.67 ± 0.21 ^a^	***
	3	1.20 ± 0.20 ^a^	2.50 ± 0.20 ^b^	4.05 ± 0.20 ^c^	5.18 ± 0.23 ^d^	2.75 ± 0.21 ^b^	***
	6	2.60 ± 0.16 ^a^	3.00 ± 0.20 ^a^	4.40 ± 0.48 ^b^	5.10 ± 0.29 ^b^	2.35 ± 0.42 ^a^	***
Rancid	0	1.00 ± 0.00	1.17 ± 0.08	1.15 ± 0.11	1.15 ± 0.08	1.13 ± 0.08	NS
	3	2.50 ± 0.35 ^b^	1.40 ± 0.19 ^a^	1.10 ± 0.07 ^a^	1.10 ± 0.10 ^a^	1.25 ± 0.11 ^a^	***
	6	1.90 ± 0.17 ^b^	1.15 ± 0.08 ^a^	1.30 ± 0.20 ^a,b^	1.20 ± 0.12 ^a^	1.05 ± 0.05 ^a^	***

**, ***: Significance level of 0.01 and 0.001, respectively. ^a,b,c,d^: Values in the same row with different superscript are significantly different (*p* < 0.05), effect of spiced added. NS: not significant.

**Table 4:** Effect of type of spice added on sensory attributes (mean ± s.e.) of cooked lamb meat burgers from Formula LNB (2/3 leg + 1/3 neck and breast meat) under aerobiosis conditions for up 0 to 6 days.

Attributes	Analysis Time	Garlic	Rosemary	Thymus	Clove	Sage	Effect of the Spice
**Odour**							
Meat	0	4.56 ± 0.23 ^b^	3.94 ± 0.1 ^b^	3.90 ± 0.19 ^b^	2.16 ± 0.28 ^a^	4.46 ± 0.19 ^b^	***
	3	4.11 ± 0.27 ^b^	3.5 ± 0.24 ^b^	3.80 ± 0.15 ^b^	2.11 ± 0.41 ^a^	4.25 ± 0.21 ^b^	***
	6	3.95 ± 0.25 ^b^	4.38 ± 0.21 ^b^	3.22 ± 0.14 ^b^	2.20 ± 0.25 ^a^	4.30 ± 0.26 ^b^	***
Spiced	0	3.03 ± 0.16 ^a^	2.66 ± 0.2 ^a^	2.83 ± 0.3 ^a,b^	4.88 ± 0.34 ^b^	2.10 ± 0.22 ^a^	***
	3	3.22 ± 0.20 ^a^	2.80 ± 0.3 ^a^	3.00 ± 0.19 ^a^	5.27 ± 0.23 ^b^	2.20 ± 0.28 ^a^	***
	6	2.85 ± 0.22 ^a,b^	2.00 ± 0.25 ^a^	3.11 ± 0.32 ^b^	4.85 ± 0.25 ^c^	2.05 ± 0.23 ^a,b^	***
Rancid	0	1.06 ± 0.06	1.05 ± 0.05	1.13 ± 0.07	1.05 ± 0.05	1.14 ± 0.09	NS
	3	1.16 ± 0.11	1.15 ± 0.10	1.15 ± 0.10	1.00 ± 0.00	1.25 ± 0.11	NS
	6	1.55 ± 0.21	1.11 ± 0.11	1.50 ± 0.20	1.25 ± 0.15	1.10 ± 0.06	NS
**Colour**							
Meat	0	5.53 ± 0.14 ^b^	5.22 ± 0.12 ^a,b^	5.16 ± 0.16 ^a,b^	4.55 ± 0.36 ^a^	5.35 ± 0.14 ^b^	*
	3	5.38 ± 0.21	5.35 ± 0.24	5.10 ± 0.16	4.55 ± 0.25	5.30 ± 0.17	NS
	6	5.50 ± 0.18 ^b^	5.16 ± 0.27 ^b^	4.94 ± 0.19 ^a,b^	4.35 ± 0.21 ^a^	5.55 ± 0.15 ^b^	***
**Flavour**							
Meat	0	4.6 ± 0.21 ^b^	4.27 ± 0.20 ^b^	3.53 ± 0.29 ^b^	2.16 ± 0.38 ^a^	4.35 ± 0.2 ^b^	***
	3	4.44 ± 0.19 ^c^	3.9 ± 0.20 ^b,c^	3.55 ± 0.20 ^b^	1.94 ± 0.31 ^a^	4.35 ± 0.18 ^c^	***
	6	4.20 ± 0.40 ^b,c^	4.00 ± 0.20 ^b,c^	2.72 ± 0.29 ^a,b^	2.15 ± 0.32 ^a^	4.60 ± 0.19 ^c^	***
Spiced	0	2.73 ± 0.20 ^a^	2.83 ± 0.20 ^a^	3.26 ± 0.29 ^a^	4.94 ± 0.30 ^b^	2.71 ± 0.19 ^a^	***
	3	2.94 ± 0.15 ^a,b^	3.20 ± 0.34 ^a,b^	3.65 ± 0.32 ^b,c^	5.16 ± 0.35 ^c^	2.25 ± 0.23 ^a^	***
	6	2.70 ± 0.26 ^a^	2.66 ± 0.40 ^a^	3.66 ± 0.32 ^b,c^	4.5 ± 0.31 ^b^	2.40 ± 0.22 ^a^	***
Rancid	0	1.40 ± 0.2	1.11 ± 0.07	1.50 ± 0.33	1.27 ± 0.14	1.32 ± 0.17	NS
	3	1.38 ± 0.18	1.05 ± 0.05	1.20 ± 0.11	1.16 ± 0.11	1.20 ± 0.11	NS
	6	1.50 ± 0.22	1.22 ± 0.14	1.61 ± 0.28	1.45 ± 0.19	1.10 ± 0.06	NS

*, ***: Significance level of 0.05 and 0.001, respectively. ^a,b,c^: Values in the same row with different superscript are significantly different (*p* < 0.05), effect of spiced added. NS: not significant.

**Table 5:** General linear model to analyse the effect of spice and formula on sensorial attributes of cooked lamb burgers.

Attributes	Spice	Formula	Spice * Formula
**Odour**			
Meaty	***	NS	**
Spiced	***	NS	NS
Rancid	***	NS	***
**Colour**			
Meaty	***	NS	NS
**Flavour**			
Meaty	***	NS	NS
Spiced	***	NS	**
Rancid	***	NS	**

Formula L: Leg lamb meat. Formula LNB: 2/3 leg + 1/3 neck and breast meat. **, ***: Significance level of 0.01 and 0.001, respectively. NS: not significant.
